# Supplementary material for: Polyester nasal swabs collected in a dry tube are a robust and inexpensive, minimal self-collection kit for SARS-CoV-2 testing
Source: PLoS One. 2021 Apr 14;16(4):e0245423. doi: 10.1371/journal.pone.0245423 (PMC8046217; doi:10.1371/journal.pone.0245423)
Supplement: S1 Text — (DOCX) [file pone.0245423.s001.docx]

## Swab physical specifications and specimen absorption measurements

An electronic digital caliper was used to measure the swab bud width and length for each swab type. To measure width, the caliper was zeroed and then extended to fit the widest portion of the swab bud. The length of the swab bud was measured from the tip of the swab bud to the stem. For each swab type, the average measurement for ten swabs was calculated.

To measure swab absorption, swabs were submerged into a 2 mL tube containing 200 µL PBS for 10 seconds (passive) and then removed or were abraded in a circular motion against the side of the tube five times in each direction and then removed. The tubes were quickly spun down and the absorption volume was calculated from the residual volume in the tube, which was measured with a micropipette.

## Quantification of SARS-CoV-2 specimen pools

SARS-CoV-2 GCE values were determined for the master positive pool, the low-positive clinical pool (2x LoD), and the high-positive clinical pool (10x LoD) using digital PCR (dPCR) and standard curve methods. For dPCR, three independent RNA extractions from the master positive pool were converted to cDNA using SuperScript IV First-Strand Synthesis System (Fisher Scientific). Following cDNA synthesis, dPCR was performed using the U.S. CDC 2019-nCoV_N2 Assay (Integrated DNA Technologies) and the QuantStudio 3D Digital PCR System to determine the number of SARS-CoV-2 *N* gene copies present in the master positive pool. The low- and high-positive clinical pool viral concentrations were below the dPCR limit of detection, therefore, the *N* gene copies were calculated based on the master positive pool *N* gene values and the dilution factors used to create the low- and high-positive clinical pools. In addition, SARS-CoV-2 viral GCE values were confirmed in the low- and high-positive clinical pools using the standard curve method. Heat inactivated 2019 Novel Coronavirus was obtained from ATCC (#VR-1986HK; lot#70035039: 3.75 x 10^5^ GCE/µL) and analyzed using the TaqPath COVID-19 Combo Kit. A 1:10 serial dilution of the inactivated ATCC viral extraction was used to generate a standard curve: 100,000 to 0.1 GCE/PCR reaction. The low- and high-positive clinical pools were tested at the same time and the GCEs were extrapolated from the standard curve.
