# Supplementary figures and images for: Polyester nasal swabs collected in a dry tube are a robust and inexpensive, minimal self-collection kit for SARS-CoV-2 testing
Source: PLoS One. 2021 Apr 14;16(4):e0245423. doi: 10.1371/journal.pone.0245423 (PMC8046217; doi:10.1371/journal.pone.0245423)

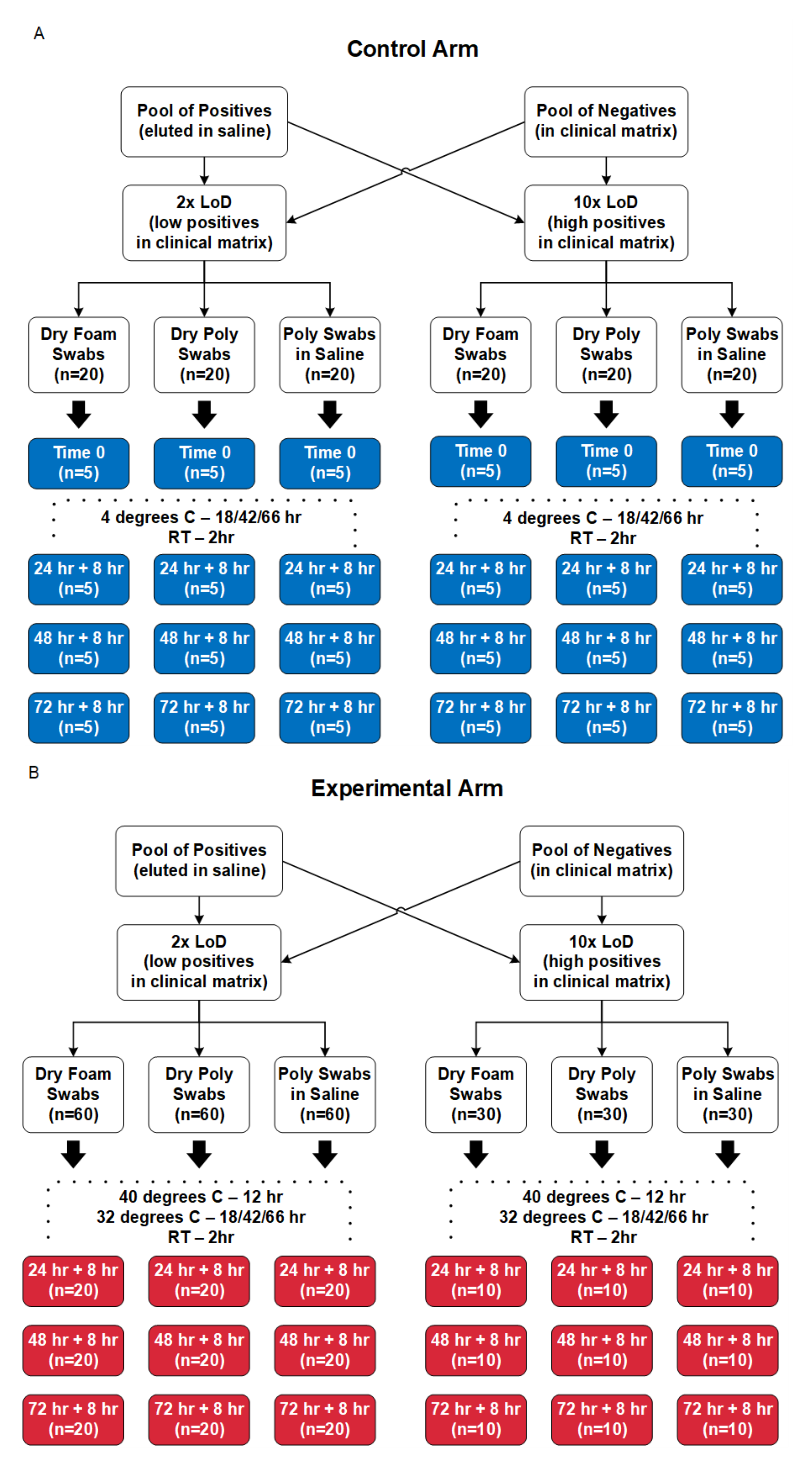

Supplement: S1 Fig — Swabs were stored with refrigeration (A, control arm) and with extended periods of time at high temperatures (B, experimental arm). Swabs spiked with the pool of negatives (n = 2 per swab type) are not shown. (TIF) [file pone.0245423.s002.tif]
