# Supplementary material for: Polyester nasal swabs collected in a dry tube are a robust and inexpensive, minimal self-collection kit for SARS-CoV-2 testing
Source: PLoS One. 2021 Apr 14;16(4):e0245423. doi: 10.1371/journal.pone.0245423 (PMC8046217; doi:10.1371/journal.pone.0245423)
Supplement: S1 Table — Mean Ct and ΔCt values (stability time point– 0 h) ± standard deviation of the high-positive (10x LoD) pool in human matrix. (DOCX) [file pone.0245423.s003.docx]

**S1 Table. Comparison of Copan and SteriPack (#60564) dry polyester swabs using human matrix to demonstrate comparability of performance and to validate stability.** Mean Ct and ΔCt values (stability time point – 0 h) ± standard deviation of the high-positive (10x LoD) pool in human matrix.

| **Target** | **Paired Swab** | **Refrigerated** | | | **Elevated Temperature** | | | |  |
| --- | --- | --- | --- | --- | --- | --- | --- | --- | --- |
|  |  | **0 h Mean Ct (n=10)** | **72 h Mean Ct (n=10)** | **ΔCt** | | **0 h Mean Ct (n=3)** | **48 h Mean Ct (n=10)** | **ΔCt** | |
| *N* | Copan | 28.4 ± 0.7 | 29.3 ± 1.6 | 0.9 ± 1.6 | | 29.4 ± 0.5 | 28.7 ± 0.7 | -0.7 ± 0.7 | |
|  | SteriPack | 28.3 ± 0.8 | 29.2 ± 1.2 | 0.9 ± 1.2 | | 29.6 ± 0.3 | 28.4 ± 0.5 | -1.1 ± 0.5 | |
| *ORF1ab* | Copan | 27.6 ± 0.7 | 28.1 ± 1.3 | 0.5 ± 1.5 | | 26.5 ± 0.5 | 28.4 ± 0.8 | 1.8 ± 0.8 | |
|  | SteriPack | 27.4 ± 0.8 | 28.1 ± 0.9 | 0.7 ± 1.1 | | 26.5 ± 0.4 | 27.9 ± 0.6 | 1.4 ± 0.6 | |
| *S* | Copan | 28.6 ± 0.9 | 28.9 ± 1.4 | 0.3 ± 1.6 | | 27.2 ± 0.3 | 27.4 ± 0.7 | 0.2 ± 0.7 | |
|  | SteriPack | 28.4 ± 1.2 | 28.8 ± 1.0 | 0.4 ± 1.4 | | 27.2 ± 0.2 | 27.1 ± 0.6 | -0.1 ± 0.6 | |
| *RNase P* | Copan | 25.4 ± 1.2 | 26.2 ± 1.2 | 0.8 ± 1.3 | | 23.8 ± 1.7 | 26.0 ± 2.3 | 2.2 ± 2.3 | |
|  | SteriPack | 25.7 ± 1.2 | 26.5 ± 1.1 | 0.8 ± 1.5 | | 24.5 ± 1.1 | 26.0 ± 1.9 | 1.5 ± 1.9 | |
