# Supplementary material for: Polyester nasal swabs collected in a dry tube are a robust and inexpensive, minimal self-collection kit for SARS-CoV-2 testing
Source: PLoS One. 2021 Apr 14;16(4):e0245423. doi: 10.1371/journal.pone.0245423 (PMC8046217; doi:10.1371/journal.pone.0245423)
Supplement: S2 Table — (DOCX) [file pone.0245423.s004.docx]

**S2 Table. Respondent characteristics for the HealthPulse@home COVID-19 Specimen Collection Kit usability studies (pediatric parent/guardian, n = 38; adult, n = 33).**

| **Characteristic** | **Pediatric** | | **Adult** | |
| --- | --- | --- | --- | --- |
|  | **n** | **%** | **n** | **%** |
| Gender (parent/guardian or adult) |  |  |  |  |
| Female | 21 | 55.3 | 17 | 51.5 |
| Male | 17 | 44.7 | 16 | 48.5 |
| Age (adult participant) |  |  |  |  |
| 18-30 years | -- | -- | 10 | 30.3 |
| 31-40 years | -- | -- | 9 | 27.3 |
| 41-54 years | -- | -- | 6 | 18.2 |
| 55-65 years | -- | -- | 4 | 12.1 |
| 66+ years | -- | -- | 4 | 12.1 |
| Age (pediatric swabbed participant) |  |  |  |  |
| 3-6 years | 8 | 21.1 | -- | -- |
| 7-10 years | 10 | 26.3 | -- | -- |
| 11-14 years | 10 | 26.3 | -- | -- |
| 15-17 years | 10 | 26.3 | -- | -- |
| Ethnicity (parent/guardian or adult) |  |  |  |  |
| Hispanic or Latino | 3 | 7.9 | 5 | 15.2 |
| Not Hispanic or Latino | 35 | 92.1 | 28 | 84.8 |
| Race (parent/guardian or adult) |  |  |  |  |
| Asian | 4 | 10.5 | 3 | 9.1 |
| Black or African-American | 9 | 23.7 | 4 | 12.1 |
| White | 21 | 55.3 | 24 | 72.7 |
| Mixed Race | 3 | 7.9 | 0 | 0 |
| Other | 1 | 2.6 | 2 | 6.1 |
| Education (parent/guardian or adult) |  |  |  |  |
| High school graduate, diploma or equivalent | 4 | 10.5 | 3 | 9.1 |
| Some college, no degree | 6 | 15.8 | 12 | 36.4 |
| Trade/technical/vocational training | 2 | 5.3 | 1 | 3.0 |
| College graduate, associate or bachelor’s degree | 19 | 50.0 | 10 | 30.3 |
| Graduate or Doctorate degree | 7 | 18.4 | 7 | 21.2 |
