# Supplementary material for: Polyester nasal swabs collected in a dry tube are a robust and inexpensive, minimal self-collection kit for SARS-CoV-2 testing
Source: PLoS One. 2021 Apr 14;16(4):e0245423. doi: 10.1371/journal.pone.0245423 (PMC8046217; doi:10.1371/journal.pone.0245423)
Supplement: Attachment — Submitted filename: PONE-21-00298.docx [file pone.0245423.s005.docx]

The manuscript describes a basic but well considered and informative study in establishing that dry nasal swab collection using polyester swabs are an acceptable method in order to support the need for alternative sampling methods given the paucity of FLOQ swabs and PPE, especially in the early phases of the COVID-19 pandemic. While the team did not use direct clinical samples, their methodology was robust and the key variants re swab types, elution methods, temperature variance and measurement of the RNAs was well drafted. I appreciated that they did not overextend their analyses on the samples but keep it relatively simple to understand.

I am bemused as to why the authors do not state that their work provided the data for the FDA to allow the sue of dry nasal swabs as a reliable specimen type for SARS -CoV 2 Rt PCR, it is in the supplemental files. It is an important piece of work and they are too modest.

Comments I have that may improve the paper would be informing the reader as to why 32.5 cycles was the Ct threshold for RNase P detection and ergo sample acceptability. Allied to this is that later when assessing the user self-collection there is at least one sample that has a CT of 35.5 and yet the authors ‘pass’ this by taking the mean, which drops the RNase P below their threshold. It is not clear to me why the mean should be used as it reads as convenience. A sample collected with an RNaseP CT of 35.5 would fail for the SARS CoV-2 targets and this should be recognized, not masked by a mean application. User error is a really big factor in why home testing is a real challenge even with something as simple as swabbing the nares. The authors detail logistical challenges re shipping within 80 hrs, per the 72 hr threshold they established, reason for the high RNase P sample should be discussed.

I found the abstract, introduction, M&Ms and results clearly written and relatively easy to follow. The discussion was written out of sequence in which data and outcomes was discussed first. I would have preferred t oread the discussion in the same order as the results, and not have the self-test component raised first.

Minor things. All materials sued including software should be reference and in the same format. In this manuscript some products have catalogue #s, others don’t and GraphPad is offered by who?
