# Supplementary material for: Polyester nasal swabs collected in a dry tube are a robust and inexpensive, minimal self-collection kit for SARS-CoV-2 testing
Source: PLoS One. 2021 Apr 14;16(4):e0245423. doi: 10.1371/journal.pone.0245423 (PMC8046217; doi:10.1371/journal.pone.0245423)
Supplement: Attachment — Submitted filename: Response to Reviewers.docx [file pone.0245423.s006.docx]

PONE-D-21-00298

Polyester nasal swabs collected in a dry tube are a robust and inexpensive, minimal self-collection kit for SARS-CoV-2 testing

**Response to Reviewers**

Reviewer #1: Collection of specimens for SARS-CoV-2 testing was a significant challenge during the early phases of the covid19 pandemic, and seems likely to remain a challenge in many countries – particularly rural Africa and South America, for at least years. Although nasopharyngeal swabbing remains a “gold standard” sample for this diagnosis, mid-turbinate, nasal and saliva samples have largely replaced NP swabbing for initial diagnosis. Early studies on nasal samples were conducted using foam swabs (Tu et al., 2020), but limitations in production have required clinicians and laboratories to utilize whatever sampling materials were available.

In addition to challenges associated with the swab itself, transport has also been an issue. Laboratories have relied on VTM, UTM, saline (buffered and unbuffered), and dry swabs, without a strong evidence base for decision making. The use of dry swabs, if justified by the evidence, enables easier specimen transport, which is a “nice to have” in the countries with strong transportation infrastructure, but which is an absolute requirement in less developed countries.

Rains and coworkers have provided a useful and significant addition to the knowledge base by comparing sampling, transport and specimen elution for several swab types. The experimental approach, relying on the use of surrogate specimens, is a reasonable approach for initial investigation; the authors have used an appropriate approach and have described the work in a manner that can be replicated. Importantly, they have used viral load levels that are relatively near the limit of detection for the RT-PCR assay employed in their study. While one might have wished the authors to compare transport with PBS, VTM, UTM and MTM, as well as sterile saline, the decision to compare only with saline is reasonable, given the number of laboratories that have utilized saline as a transport medium, and the lack of data suggesting significant superiority of any other transport medium.

The inclusion of freeze-thaw stability studies and high temperature stability studies is particularly noteworthy, since both may apply to rural areas with extreme environments (Northern US and Canada, mountains, rural South America, US and Africa).

I have several rather minor critiques of the manuscript. It is not obvious to me that the average reader will understand the clinical importance of the Table 1 data on absorption – I recommend that the authors provide some explanation.

We thank the Reviewer for the comment. We have expanded the sentence to include clinical relevance.

“Polyester swabs were 150% more absorbent than foam swabs by the swirling method and by passive absorption (Table 1) and thus, polyester swabs may be advantageous in the clinical setting as they contain a larger sample capacity.”

I also believe that this manuscript would benefit by a statement of limitations in the study. An ideal study would compare dry collection and transport for spun polyester with a reference technique, such as the nasal swab or NP swab method reported by Tu et al (Tu et al., 2020), using a similar study design. Such a study would provide more confidence in the results, and I think it would be worth pointing this out in the manuscript. I have not attempted to think through a comprehensive list of limitations, but I am sure that the authors would have others to add.

We agree with the reviewer and we have added a statement of limitations paragraph in the discussion (lines 464-475).

This is a significant contribution.

Tu, Y.-P. P., Jennings, R., Hart, B., Cangelosi, G. G. A., Wood, R. C., Wehber, K., Verma, P., Vojta, D., & Berke, E. M. (2020). Swabs collected by patients or health care workers for SARS-CoV-2 testing. The New England Journal of Medicine, 383(5), 494–496. https://doi.org/10.1056/NEJMc2016321

Reviewer #2: The manuscript describes a basic but well considered and informative study in establishing that dry nasal swab collection using polyester swabs are an acceptable method in order to support the need for alternative sampling methods given the paucity of FLOQ swabs and PPE, especially in the early phases of the COVID-19 pandemic. While the team did not use direct clinical samples, their methodology was robust and the key variants re swab types, elution methods, temperature variance and measurement of the RNAs was well drafted. I appreciated that they did not overextend their analyses on the samples but kept it relatively simple to understand.

I am bemused as to why the authors do not state that their work provided the data for the FDA to allow the use of dry nasal swabs as a reliable specimen type for SARS -CoV 2 Rt PCR, it is in the supplemental files. It is an important piece of work and they are too modest.

We thank the reviewer for pointing out the significance of this work. We have moved the paragraph describing the significance of the data from the supplemental files to the Results section (lines 341-348). We also added the following sentence to the Discussion. “Our studies provided the FDA to allow the use of foam or polyester nasal swabs transported in saline, PBS, or dry tubes as a reliable specimen type for SARS-CoV-2 detection by RT-qPCR and this data has been made available via a Right of Reference.”

Comments I have that may improve the paper would be informing the reader as to why 32.5 cycles was the Ct threshold for RNase P detection and ergo sample acceptability?

We agree with the reviewer’s comment and have updated the sentence to improve clarity. While we stated that the mean 32 cycle threshold was used as a cutoff in the methods, we failed to add that detail to the text. Additionally, this value was determined through discussions with the FDA. We have updated the following sentences in the methods and results.

“The acceptance criteria for specimen collection within each study population was a mean RNase P Ct value of ≤ 32 across all specimens (based on conversations with FDA during informal pre-submission discussions regarding emergency use authorization for the Audere home collection kit).

“The predefined threshold (mean ≤ 32 cycles as required by the FDA) for nucleic acid sufficiency was met with mean RNase P Ct values of 23.4 and 25.1 in the adult and pediatric studies, respectively.”

Allied to this is that later when assessing the user self-collection there is at least one sample that has a CT of 35.5 and yet the authors ‘pass’ this by taking the mean, which drops the RNase P below their threshold. It is not clear to me why the mean should be used as it reads as convenience to me. A sample collected with an RNaseP CT of 35.5 would fail for the SARS CoV-2 targets and this should be recognized, not masked by an application of the mean Ct. User error is a really big factor in why home testing is a real challenge even with something as simple as swabbing the nares. The authors detail logistical challenges re shipping within 80 hrs, per the 72 hr threshold they established, reason for the high RNase P sample should be discussed.

We agree with the reviewer’s comment and address this in the statement of limitations paragraph.

“A second limitation of this study is that the usability studies comprised of only healthy individuals and *RNase P* testing. Of note, one sample in the adult usability study had a *RNase P* value of 35.5 and while the overall study met the FDA defined mean ≤ 32 cycle threshold, it is possible that this sample would fail SARS-CoV-2 detection. The high RNase P value is likely due to user variability which is a significant challenge in home collection. Further studies are needed to assess home-based nasal specimen self-collection, particularly in SARS-CoV-2 positive individuals, and to improve overall home collection processes.”

I found the abstract, introduction, M&Ms and results clearly written and relatively easy to follow. The discussion was written out of sequence in terms of which data and outcomes was discussed first. I would have preferred to read the discussion in the same order as the results, and not have the self-test component raised first.

We have rearranged the discussion to align with the results section.

Minor things. All materials used including software should be reference and in the same basic format. In this manuscript some products have catalogue #s, others don’t and GraphPad is offered by who?

We have updated the manuscript to include the additional details for the software. The basic material reference format includes only the manufacturer name, which is sufficient for the general laboratory materials. However, for the swabs, we feel it is necessary to include the item #s for clarity.
